# Utilizing the intelligent sensing function of conjugated materials to create intelligent flute training equipment

**DOI:** 10.3389/fchem.2023.1301656

**Published:** 2023-11-09

**Authors:** Jialiang Lu

**Affiliations:** Personnel Department, Shanxi Vocational College of Art, Taiyuan, Shanxi, China

**Keywords:** conjugated material, intelligent sensing function, flute training equipment, flexible electronics, photoelectric conversion

## Abstract

The manufacturing of flutes has always relied on traditional ceramics and metals, which may be affected by various factors during the manufacturing process, as well as the lack of intelligent sensing functions, resulting in poor sound quality and performance of the instrument. The purpose of this article is to explore the use of the intelligent sensing function of conjugated materials to create intelligent flute training equipment, achieve automatic tuning, volume control, etc., and improve the playing experience and training effect of the instrument. This article first analyzes the smart sensing function of conjugate materials and applies it to smart flute training equipment; then, introduces photosensitive materials at appropriate locations to change the size and shape of the flute’s sound hole, thereby adjusting the timbre; finally, uses smart flute training based on conjugate materials for real-time perception of performers’ performance experiments. The test results show that the average delay time of the conjugate material trench is reduced by 73.1% compared with the average delay time of the ceramic trench, and is reduced by 63.5% compared with the average delay time of the metal trench. This shows that the conjugated material flute is more intelligent and can quickly respond to the player’s performance and automatically control and respond.

## 1 Introduction

Flute is a professional instrument, an art, and a music discipline. Flute training requires music students to practice and persist for a long time in order to acquire good performance skills and expressive abilities. However, in traditional flute practice, the use of equipment brings many challenges and limitations, such as difficulty in maintenance and adjustment. Learners often rely on feedback from mentors and their own feelings in order to perform correctly. This method is not always effective, and sometimes it cannot determine some minor technical issues. Conjugated materials are widely used due to their special properties. Applying conjugated materials to flute training equipment can achieve real-time perception of students’ playing movements, respiratory control, and pitch information, thereby achieving the goal of improving performance.

In order to study how to integrate vibrato into the timbre of flute for better training and performance, Zheng and Charoensloong (2022) analyzed the current perspectives of the flute academic community and conducted academic analysis and critical discussions. He verified the scientificity and effectiveness of their respective teaching methods, and critically summarized and analyzed the flute vibrato technology and teaching. In order to create true smooth notes, Yonce Tammy (2018) proposed using smooth joints to greatly expand the expressive power of the flute. When the joints are in the “main position,” the sound emitted by the flute is exactly like a standard performer’s playing section. In various professional fields of music, they do not exist in isolation. In order to understand the commonalities between flute performance and vocal music, Yuan (2019) analyzed the training of flute timbre and the commonalities with vocal music production methods from three aspects: ear training, breathing training, and throat skill training, aiming to improve the efficiency and effectiveness of flute timbre training. The above scholars have found that traditional flute training is generally completed through theoretical guidance or tools such as connectors, and does not have the characteristics of intelligence. To make flute training more flexible and convenient, it is necessary to start by changing its material, such as the popular conjugated material.

When learners play inaccurately or with unstable rhythms, intelligent flute training equipment can sense through conjugated materials. Traditional flute equipment does not have the characteristics of intelligence. Zhang et al. (2023) proposed the emerging conjugated polymer semiconductor. The electromechanical chemical transistor made of conjugated polymers has become a research hotspot due to its easy preparation, ion electron conversion ability, and biological interface compatibility. This is an effective solution and meets the usage needs of flexible equipment, greatly promoting the application and development of flute equipment. In order to explore the mechanical properties of SiO2 composites, Wang et al. (2018) prepared SiO2 composites by sol-gel method, and then prepared hybrid materials by melt blending with polypropylene. The mechanical performance test results confirmed that it has a good synergistic modification effect of strengthening and toughening, and is superior to hybrid material systems. Gong (2018) synthesized a diatomaceous earth polypropylene composite material with excellent sound absorption performance using diatomaceous earth, polypropylene, foaming agent, and porous agent as precursor materials. He evaluated the sound absorption performance of composite materials using a transfer function impedance tube sound absorption testing system. The results indicated that it has excellent compressibility, with a sound absorption coefficient of 0.85. The above scholars believe that the introduction of intelligent training equipment based on conjugated materials can provide real-time and accurate feedback, help learners better understand and master performance skills, and better present the emotions and artistic conception that music works are intended to express.

This article analyzed intelligent flute training equipment using the intelligent sensing function of conjugated materials to enhance the effectiveness and experience of flute learning and training. In flute performance, the blowing force and fingering technique of the performer are important factors that affect the timbre and volume of the performance. This article applied conjugated materials to flexible sensors, which could sense changes in blowing force and fingering in real-time. Flexible sensors could be placed in appropriate positions in the flute to sense the intensity and direction of airflow. The preparation of flexible electronic equipment using conjugated materials is an important aspect of studying their electronic transmission and electronic equipment performance. By changing the blowing intensity of the performer, corresponding electrical signals were generated at different blowing intensities, and processed to determine the blowing intensity of the performer. Based on the required acoustic model, different tone quality and volume were achieved according to different playing methods. The introduction of photoelectric sensing technology based on conjugated materials into flute training equipment helps to improve the performer’s performance level and provides a more intelligent and efficient method for flute manufacturing.

## 2 Conjugated materials

**FIGURE 1:**
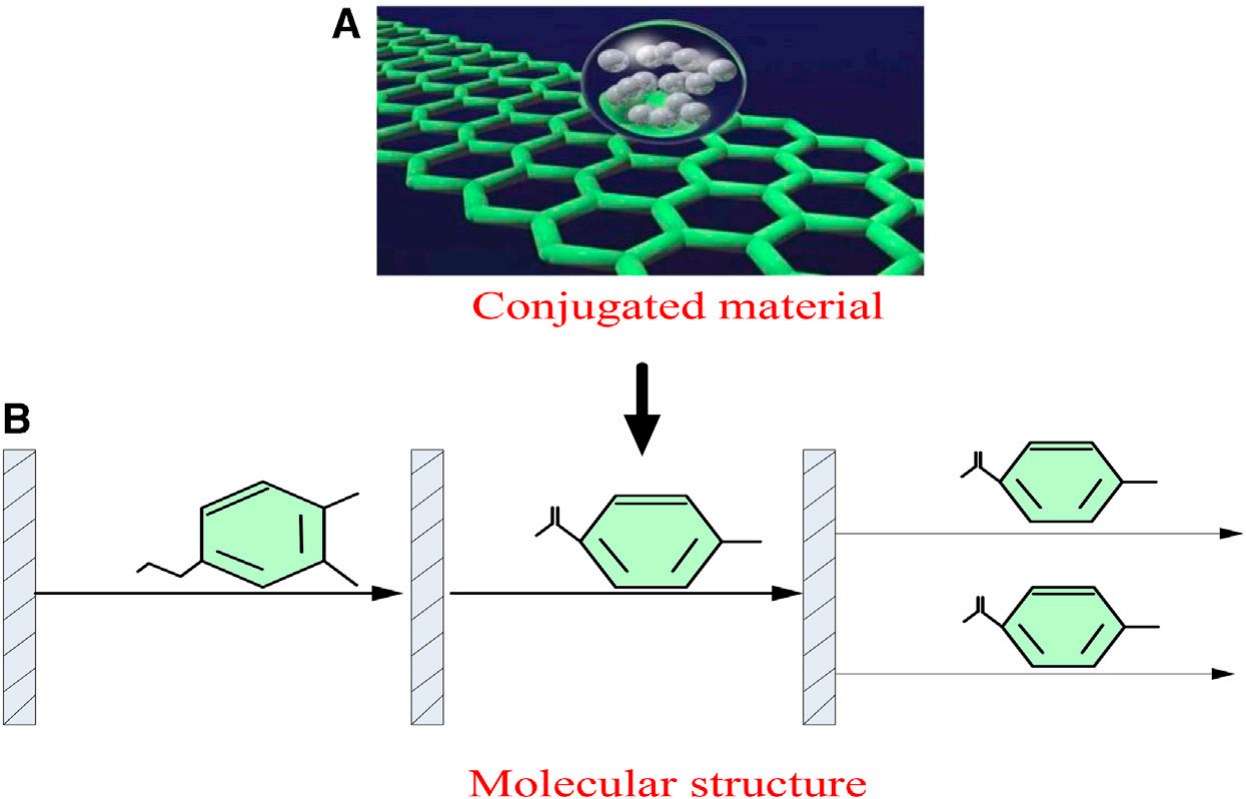
Schematic diagram of conjugated materials. **(A)** Conjugated material. **(B)** Molecular structure.

Conjugated materials are a special type of material with special structures and properties that can convert external stimuli into electrical signals or other forms of signals. The special structure of this material gives it good conductivity and sensing function (Li et al., 2019; Shao, 2019). The schematic diagram of the conjugated material is shown in Figure 1.

As shown in Figure 1: a is the conjugated material, and b is the molecular structure of the conjugated material.

The structure of conjugated materials is composed of conjugated polymers, which refer to polymers in which π electrons extend in a conjugated manner to form a continuous π electron conjugation system. This conjugated system can be achieved by sharing π electron orbitals that overlap with adjacent monomer molecules, and the structure of conjugated polymers can also be achieved by controlling the topological structure of the conjugated system. The continuous π electron system of one-dimensional conjugated polymers is usually arranged in a plane, forming a parallel stacked ordered structure. This structure facilitates the transport of electrons in the conjugated system and can adjust optical and electronic properties by adjusting the spacing and interaction between chains.

### 2.1 Functional adjustability

Conjugated materials, due to their unique electronic structure and energy level distribution, can exhibit excellent photoelectric properties. Through material design and synthesis regulation, conjugated materials can have specific optical, electrical, or thermal properties.

In terms of optics, conjugated materials can have high absorption and emission properties, as well as longer photoelectron lifetime. This can be achieved by designing the conjugated structure reasonably and introducing different functional groups and conjugated units. Introducing electron withdrawing or conducting groups into the molecular structure can adjust the band gap and energy level structure of conjugated materials, thereby changing their absorption and emission wavelengths. In addition, by adjusting factors such as the conjugated length of molecules, the spatial arrangement of functional groups, and the orientation of molecules, the nonlinear optical properties of conjugated materials can also be adjusted, such as second harmonic generation and optical limiting (Li, 2021).

The relationship between the bandgap energy of conjugated materials and the maximum absorption wavelength is used to predict the bandgap energy of conjugated materials, and the formula is:

$$E_{g}=\frac{h_{c}}{\lambda_{\max}}$$ (1)

The bandgap energy $E_{g}$ represents the spacing of the conjugated material, and $\lambda_{\max}$ represents the maximum absorption wavelength of the material. This is a simplified relationship that is crucial for the performance of photoelectric equipment and can directly affect the photoelectric conversion efficiency and light absorption capacity.

In terms of electricity, conjugated materials can have excellent carrier transport performance and charge mobility. The molecular structure of conjugated materials can adjust their energy level distribution, thereby affecting the transport behavior of charge carriers. The filling factor is a parameter that measures the performance of photoelectric equipment, representing the proportion of the rectangular area of the current-voltage characteristic curve to its maximum possible value:

$$G_{F}=\frac{\left(J_{\text{mp}}\times V_{\text{mp}}\right)}{J_{\text{sc}}\times V_{\text{oc}}}$$ (2)

$G_{F}$ represents the filling factor; $J_{\text{mp}}$ represents the photocurrent density at the maximum power point; $V_{\text{mp}}$ represents the voltage at the maximum power point. A higher fill factor value indicates that photoelectric equipment have better current transmission and charge collection capabilities, which is beneficial for improving equipment efficiency.

By adjusting the highest occupied molecular orbital and lowest unoccupied molecular orbital energy levels of materials, the mobility of charge carriers and charge injection efficiency can be improved, thereby improving the performance of equipment. The highest occupied molecular orbital refers to the orbital with the highest energy level of occupied electrons during a chemical reaction. In addition, by introducing auxiliary electron donors or acceptor groups, the infrared, visible spectrum, and electronic structure of conjugated materials can also be adjusted to achieve optimization of photoelectric conversion equipment.

In terms of thermodynamics, conjugated materials can have excellent thermal stability and thermal conductivity. The chain structure and molecular arrangement of conjugated materials can increase their intermolecular interactions, and improve the melting point and thermal stability of molecules. In addition, introducing functional groups with good thermal conductivity into conjugated materials can improve their thermal conductivity and reduce the thermal failure problem of equipment.

### 2.2 Photovoltaic conversion

Conjugated materials are a type of organic or inorganic materials with special electronic structures, which can exhibit excellent photoelectric conversion performance. When light energy is absorbed, electrons in conjugated material molecules can be excited to high energy states, forming electron-hole pairs. Then, through charge separation and transmission processes, light energy is converted into electrical energy or other forms of energy. Electron-hole pair refers to the process of using photons with energy equal to or greater than the bandgap to irradiate a semiconductor, where photons are absorbed and excite electrons in the valence band into the conduction band, forming a hole in the valence band, that is, producing excess or balanced carriers.

Conjugated materials have a special electronic structure, where there is a conjugated system that can absorb photons and cause electrons within the molecule to transition to high-energy excited states. When conjugated materials are exposed to external light sources, their absorbed energy matches the energy of photons, and electrons are excited and enter the excited state.

**FIGURE 2:**
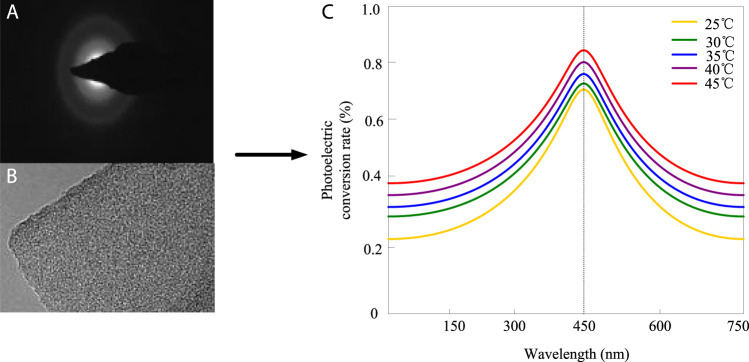
Analysis of photoelectric conversion. **(A)** Lighting status. **(B)** State with electrons. **(C)** Photoelectric conversion situation.

The photoelectric conversion analysis of conjugated materials at different wavelengths (150, 300, 450, 600, and 750 nm) at different endothermic reaction temperatures (25°C, 30°C, 35°C, 40°C, 45°C) is shown in Figure 2 (the abscissa of Figure 2C represents the wavelength in nm, and the ordinate represents the photoelectric conversion rate).

Figure 2A is the state of light; Figure 2B is the state with electrons; Figure 2C is the case of photoelectric conversion. The temperature is the endothermic reaction temperature, with the highest conversion rate at a wavelength of around 450 nm.

In conjugated materials, excitons formed by light excitation undergo an electron separation process, which effectively separates electrons and holes, preventing recombination. This process typically occurs at interfaces or heterostructures within materials, where one material with a higher energy level captures electrons and another material with a lower energy level captures holes. The photoelectric conversion efficiency is an important indicator for evaluating the performance of photoelectric conversion materials, representing the efficiency of converting light energy into electrical energy. The calculation formula is as follows:

$$P_{\text{CE}}=\frac{\left(J_{\text{sc}}\times V_{\text{oc}}\times G_{F}\right)}{P_{\text{in}}}$$ (3)

$J_{\text{sc}}$ represents the short-circuit current density of the photocell; $V_{\text{oc}}$ represents open circuit voltage; $P_{\text{in}}$ represents the power density of the incident light.

In conjugated materials, excitons formed by light excitation undergo an electron separation process, which effectively separates electrons and holes, preventing recombination. The photoelectric conversion efficiency is an important indicator for evaluating the performance of photoelectric conversion materials, representing the efficiency of converting light energy into electrical energy. The calculation formula is as follows:

$$Q_{E}=\frac{E_{L}}{P_{H}}$$ (4)

Among them, $E_{L}$ represents the number of electrons obtained from the photocell per unit time, and $P_{H}$ represents the number of photons incident into the photocell per unit time.

During this process, electrons and holes are transferred in the form of charges between the positive and negative electrodes, generating current and outputting it in the form of electrical energy. In photoelectric equipment such as solar cells, the electrodes used are generally conductive materials such as metals or conductive polymers, which can efficiently sense the electrons and holes entering them and convert them into usable electrical energy. Its optical absorption spectrum, band structure, electron affinity, and electron mobility all affect its photoelectric conversion properties.

Therefore, in practical design and application, it is necessary to select appropriate conjugated materials based on specific application requirements, and optimize the structure and properties of the materials to achieve more efficient photoelectric conversion effects.

## 3 Design of intelligent flute training equipment

### 3.1 Equipment requirements

In terms of user needs, it is first necessary to understand the level of flute learners. Flute training equipment may be aimed at beginners, advanced students, or professional performers, and each group has different needs (Sakin, 2018). Beginners may need a simple and easy-to-use interface and teaching features to make it easier for them to start learning flute, while advanced learners may need more advanced features such as practicing music display and evaluation to improve their performance level.

In order to reduce the differences in the experiment, this article selects 400 advanced users for analysis and conducts a questionnaire survey to understand the needs and preferences of the 400 advanced users. The needs and preferences of 400 flute users are shown in Table 1.

**TABLE 1:** User needs and preferences (multiple choices).

Demand	Number of people	Percentage (%)	Preference	Number of people	Percentage (%)
Intelligent	351	87.75	Brand	155	38.75
Price	176	44.00	Lightweight and portable	256	64.00
Experience	268	67.00	Material quality	321	80.25
Service	155	38.75	Appearance	289	72.25
Tone quality	309	77.25	Attachment	102	25.50

As shown in Table 1, users have the highest demand for intelligence, with a percentage of 87.75%, followed by tone quality, with a percentage of 77.25%. User preference is the highest for material quality, with a percentage of 80.25%.

It can be seen that users need more intelligent flutes. Professional performers may need more specialized and personalized training tools, and in addition to intelligence, users also hope that the equipment can provide good tone quality functions. The true reproduction of tone quality is very important for performers, so equipment needs to have high fidelity audio output capabilities.

It is also necessary to consider the competitive situation and differentiated requirements of flute training equipment to understand the existing products and services in the market, analyze their advantages and disadvantages, and identify innovative points that can meet user pain points.

### 3.2 Utilization in flute training equipment

#### 3.2.1 Photosensitive material

In conjugated materials, certain specific organic molecules or polymers undergo structural changes under the stimulation of light, and this photo induced deformation characteristic can be applied to the regulation of the sound hole of flute (Yu et al., 2022). The photosensitive substance is placed in a suitable area. Under the irradiation of light, the form of photosensitive substances changes, thereby changing the size and shape of the sound hole of the flute, achieving adjustment of the tone quality and volume of the flute. This provides performers with more space and opportunities for performance (RocheHueberGarnier et al., 2021).

Organic photosensitive materials such as polymers and organic molecular compounds are gradually being applied in the field of photoelectric sensors. These materials have high flexibility and plasticity, and can be adjusted to adjust the spectral response range and sensitivity by adjusting the chemical structure. Due to the absorption of light energy by conjugated materials, their electrons are transformed from the ground state to the excited state, resulting in the generation of excitons. In terms of photo induced deformation, the appearance of excitons after photo induced deformation leads to structural changes in the molecules in the polymer. This structural change can be described by the following formula:

ΔL=S×α×ΔT (5)

Among them, ΔL is the change in the length of the conjugated material, and S is the sensitivity of the conjugated material. α is the coefficient of thermal expansion, and ΔT is the temperature change. At a certain position, light shining on the photosensitive material can cause a local temperature change of the photosensitive material, thereby causing structural deformation of the photosensitive material. Through this structural change, the adjustment of the tone quality and volume of the flute can be achieved by changing the aperture and shape of the flute.

The photo induced deformation properties of conjugated materials can be used to control the sound hole of flute, which brings new possibilities for flute production. Photoinduced deformation mainly comes from the action of excitons in conjugated systems. During this process, the interaction between interfaces causes external stress on the interface, which in turn leads to interface deformation. This process can be represented by the following formula:

F=k×ΔE (6)

F represents external stress; k is the elastic constant; ΔE is the electric field gradient generated by excitons. By adjusting the placement position and lighting conditions of photosensitive materials, the size of the sound hole on the flute can be accurately adjusted. By fine-tuning the position and brightness of the light source, players can adjust the shape and size of the sound hole according to their own requirements, thereby achieving the goal of controlling tone quality and volume.

#### 3.2.2 Optical induction

Due to its unique band structure and electronic structure, conjugated materials can absorb light and excite a large number of excitons, thereby altering their electrical, optical, and other properties, making them more sensitive to the external environment. Due to its excellent optical properties, it has broad application prospects in optical sensing.

By utilizing the photoelectric sensor of the conjugated body, real-time detection of the position and force of the player’s mouth and fingers can be carried out in the flute practice equipment, and accurate feedback can be provided based on the detected information, thereby achieving the goal of improving the playing level. Sensors can be used to detect airflow and pressure in pipelines, thereby detecting whether the performer can play evenly.

The change in photoconductivity can be used to describe the relationship between the conductivity of conjugated materials and temperature, represented by Formula 7:

Δσ=σ×S×α×ΔT (7)

Among them, Δσ represents the change in conductivity caused by lighting, and σ represents the conductivity of the material. The change in conductivity can be used to calculate the magnitude of photo induced conductivity changes.

The piezoresistive effect refers to the phenomenon where a semiconductor undergoes a change in its electrical resistivity due to the change in energy band caused by stress, resulting in the movement of energy provided. The formula is:

ΔR=R×β×ΔI (8)

Among them, ΔR represents the change in resistance caused by lighting; β is the photoinduced piezoresistive coefficient; ΔI represents the change in current.

The conjugated material has a planar structure, which enables electrons to have good charge transport properties. Due to the coherent formation of electrons in their molecules, known as the “electron cloud,” the movement of electrons is limited by the distribution of this “electron cloud.” Therefore, common materials are an important class of semiconductor materials (Nurjanah and Mishbahatul, 2019; Park, 2019). In addition, common materials also have other excellent properties, such as good photoelectric conversion efficiency, stability, and adjustability, and can be designed with different molecular structures according to different needs.

#### 3.2.3 Flexible electronics

Conjugated materials are widely used in the field of flexible electronics, and their flexible and bendable properties make them suitable for various curved surfaces and shapes of equipment components. Flexible electronic equipment is a new electronic technology that is more adaptable to harsh working conditions (such as mechanical deformation, high and low temperatures, etc.) compared to traditional electronic equipment. Flexible electronic equipment has broad application prospects due to their high flexibility, deformability, ability to process into free form surfaces, ultra-thin shapes, and other characteristics, such as wearable electronic products, intelligent medical equipment, automotive electronics, home safety, flexible sensors, etc. With the progress of flexible electronics in materials, processes, equipment, and the demand for wearability and personalization, flexible electronics play an increasingly important role in future development (Sakin, 2021).

In flexible electronics, the resistivity of conjugated materials is an important parameter because it affects the transmission of current and the conductivity of the material. Resistivity is a physical quantity that describes the degree of material resistance, and the resistivity formula is:

$$\rho =R\times \frac{A}{L}$$ (9)

The resistivity of ρ is proportional to the resistance of R .

The rapid development of intelligent perception technology has not only greatly promoted the development of industrial interconnection and intelligent manufacturing, but also laid the foundation for many cutting-edge fields such as intelligent wearability, autonomous driving, virtual reality, and so on. The sensitivity of sensors is getting higher; the resolution is getting smaller; the detection limit is getting wider; the response speed is getting faster; the intelligence is getting smaller; there are more functions; the integration is getting stronger; the wearability is getting stronger. Conjugated materials are currently known as a new generation of intelligent materials, which not only have unique responses to light, electricity, magnetism, etc., but also have outstanding advantages such as a wide range of raw materials, simple processing, and controllable conductivity.

Conductivity refers to the electrical conductivity per unit cross-sectional area of a substance, usually measured in units of Siemens per meter. It is one of the physical quantities that describe the conductivity of a substance, reflecting the degree to which the substance conducts electricity:

$$\sigma =\frac{1}{\rho }$$ (10)

Conductivity represents the conductivity of a substance to current. The higher the conductivity, the stronger the conductivity of the substance to current, which means it can make current flow more easily. By utilizing changes in electrical resistivity, information on the movements of the performer’s fingers can be obtained, thereby achieving the goal of controlling timbre, volume, etc.

Flexible sensors and variable capacitor components composed of conjugates can be connected to computers and other data processing equipment for real-time data transmission and analysis. By analyzing information such as blowing force, fingering, and posture, feedback and guidance can be provided to performers, helping them improve their playing skills and performance (FischerSodenThoret and Montrey, 2021).

## 4 Effect of intelligent flute training equipment

The experimental objective of this article is to evaluate the effectiveness of intelligent flute training equipment in improving learners’ performance skills. Due to limited energy, only 30 out of 400 advanced performers were invited as experimental performers. The age range of performers is between 18 and 30 years old, with a certain foundation in flute playing and the ability to independently complete experimental tasks. The equipment used in the experiment includes ceramic flutes, metal flutes, and conjugated flutes.

### 4.1 Accuracy

Evaluating the performance of equipment in guiding pitch accuracy can compare differences in pitch. A smaller difference indicates that the equipment has higher pitch accuracy, and the unit of pitch is Hertz (Hz).

#### 4.1.1 Intonation accuracy

Six pieces of music were selected. Firstly, six of the performers used flutes of different materials to play, with each player playing one piece of music. The pitch errors of ceramic flutes, metal flutes, and conjugated material flutes are shown in Table 2 (pitch error is the actual pitch minus the pitch of different material flutes).

**TABLE 2:** Pitch error of flutes made of different materials.

Music	Actual pitch	Ceramic		Metal		Conjugated material	
		Pitch	Pitch error	Pitch	Pitch error	Pitch	Pitch error
1	282	276	6	272	10	280	2
2	319	305	14	310	9	319	0
3	265	221	44	241	24	264	1
4	234	209	25	220	14	233	1
5	277	258	19	261	16	275	2
6	234	217	17	226	8	232	2

As shown in Table 2, the highest pitch errors for ceramic flutes, metal flutes, and conjugated material flutes were 44, 24, and 2 Hz, respectively.

Compared to ceramics and metals, conjugated materials had lower internal losses and frequency attenuation, resulting in higher accuracy in intonation and more accurate frequency of notes.

The high conductivity and thermal conductivity of conjugated materials enable vibration to be transmitted more effectively. Conjugated material flute equipment can more accurately reproduce the player’s playing motion and vibration behavior, thereby improving the player’s pitch accuracy. This material has a wide frequency response range and can effectively transmit notes and timbre in the flute. Ceramics, metals, and other materials may exhibit resonance, attenuation, and other phenomena in certain frequency bands, thereby affecting the accuracy of pitch.

#### 4.1.2 Rhythmic accuracy

**FIGURE 3:**
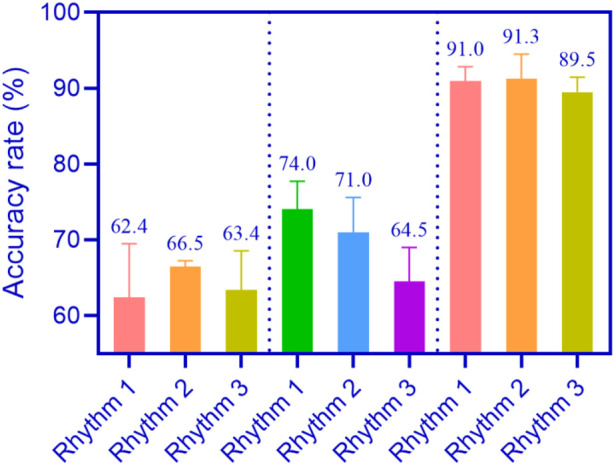
Average rhythm accuracy of flutes made of different materials.

The differences in rhythm were compared. A smaller difference indicates that the equipment has higher rhythm accuracy. Each piece of music was divided into three rhythms (prelude, climax, and ending), namely, rhythm 1, rhythm 2, and rhythm 3. The average rhythm accuracy of ceramic flutes, metal flutes, and conjugated material flutes is shown in Figure 3 (the horizontal axis of Figure 5 represents rhythm 1, rhythm 2, and rhythm 3, and the vertical axis represents accuracy).

As shown in Figure 3: Figure 3 was divided into three parts. The first part showed the average rhythm accuracy of rhythm 1, rhythm 2, and rhythm 3 in playing music using ceramic flute, which were 62.4%, 66.5%, and 63.4%, respectively. The second part was about using metal flute to perform music with an average rhythm accuracy of 74.0%, 71.0%, and 64.5% for rhythm 1, rhythm 2, and rhythm 3, respectively. The third part was about using conjugated materials to perform flute music with an average rhythm accuracy of 91.0%, 91.3%, and 89.5% for rhythm 1, rhythm 2, and rhythm 3, respectively.

The average rhythm accuracy of using conjugated materials for flute playing music is higher, because conjugated materials can better control vibration and resonance behavior, help maintain note stability, and improve the rhythm accuracy of the equipment.

Conjugated materials have the advantages of easy processing, forming, and manufacturing, which enable more accurate control of parameters such as the shape, wall thickness, and nozzle size of the flute, thereby improving the tone quality of the flute. This material has strong resistance to external interference, such as temperature, humidity, and pressure. In different working environments, conjugated materials can better maintain their physical properties without adversely affecting the accuracy of rhythm.

### 4.2 Playing error rate

**FIGURE 4:**
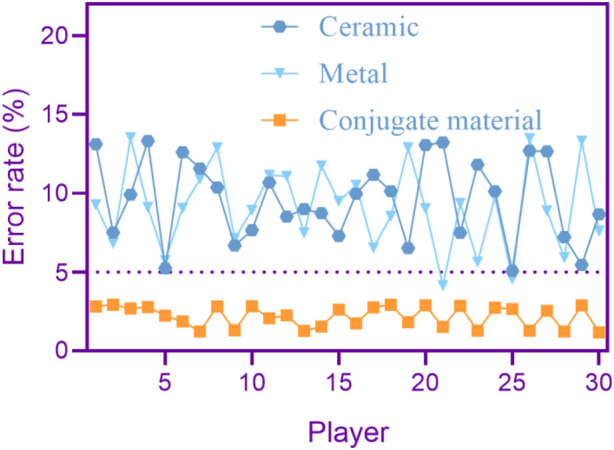
Playing error rate of flutes made of different materials.

A high error rate in playing means that the equipment’s playing effect is poor and prone to errors. In order to make the experiment more convincing, this article asked 30 performers to play the same music using ceramic flutes, metal flutes, and conjugated material flutes once, in order to distinguish which material of flute has a higher error rate in playing. The error rate of playing different materials of flutes is shown in Figure 4 (the horizontal axis in Figure 6 represents the performer, and the vertical axis represents the error rate).

As shown in Figure 4, the error rate in using ceramic and metal flutes was mostly above 5%. The error rate in using conjugated material flutes was generally within 5%, and the error rate in using conjugated material flutes was relatively stable.

The playing error rate of conjugated material flutes is lower than that of ceramic and metal flutes. Conjugated materials have better audio transmission accuracy and can more accurately transmit the performer’s audio signal and subtle changes.

### 4.3 Sensitivity

Sensitivity was evaluated through time delay. A lower time delay indicates a higher sensitivity. The time delays of ceramic flutes, metal flutes, and conjugated material flutes are shown in Table 3.

**TABLE 3:** Time delay of flutes made of different materials (ms).

Music sequence number and mean value	Ceramic	Metal	Conjugated material
1	31.85	24.49	7.51
2	25.69	19.02	5.04
3	32.29	21.96	9.34
4	25.73	21.08	8.70
5	27.42	19.56	8.58
6	31.61	22.54	7.83
Mean value	29.10	21.44	7.83

As shown in Table 3, the time delays of ceramic flutes were all above 20 ms; metal flutes were all above 15 ms; conjugated material flutes were all within 10 ms. The average time delays of ceramic flute, metal flute, and conjugated material flute were 29.10, 21.44, and 7.83 ms, respectively. The average time delay of conjugated material flutes increased by −73.1% ( $\frac{7.83-29.10}{29.10}=-73.1\%$ ) compared to ceramic flutes, and the average time delay of conjugated material flutes increased by −63.5% ( $\frac{7.83-21.44}{21.44}=-63.5\%$ ) compared to metal flutes, which was a decrease of 63.5%.

Ceramic and metal materials are relatively hard and lack elasticity, which means that they cannot respond and vibrate as quickly as conjugated materials during performance, resulting in longer time delays.

Due to the high hardness and brittleness of ceramic materials, they are more prone to fracture under external forces rather than elastic deformation. Therefore, the flute made of ceramic flute, regardless of the strength of the blowing or sound, cannot produce enough deformation to achieve the purpose of transmitting signals, which greatly limits the tone quality of ceramic flute.

Although metal materials have good conductivity and mechanical properties, they have high rigidity and lack flexibility and deformability, which makes it relatively difficult for metal flutes to respond to small changes in the player’s blowing force and fingering. In addition, metal flutes also have the limitation of high resonance frequencies, which may result in some weak signals being masked or missing, thereby reducing sensitivity.

### 4.4 Training effectiveness

**FIGURE 5:**
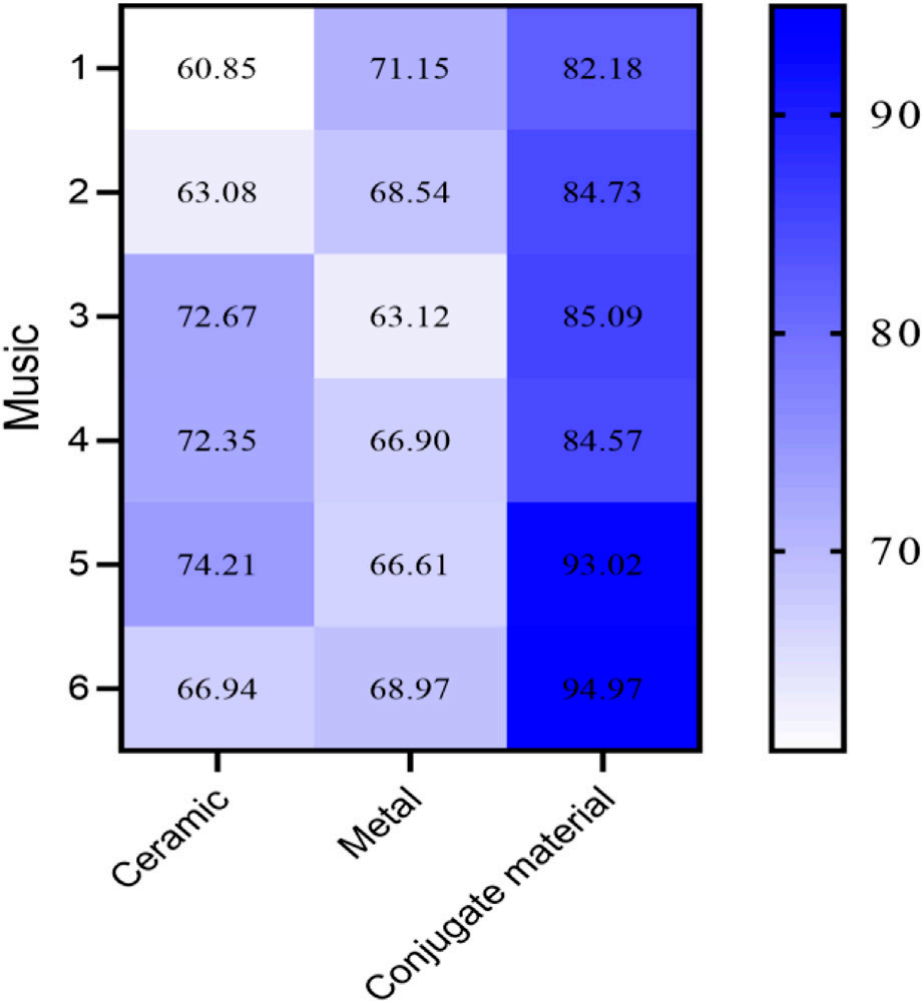
Scoring of ceramic flute, metal flute, and conjugated material flute.

Thirty performers were asked to play 6 different pieces of music using flutes made of different materials, and the scores were given. The average scores of ceramic, metal, and conjugated material flutes are shown in Figure 5 (in Figure 5, the horizontal axis represents the flutes of ceramic, metal, and conjugated material, and the vertical axis represents music. The data in Figure 5 has been averaged).

As shown in Figure 5, the performance score of ceramic and metal flutes was below 80 points, while the performance score of conjugated material flutes was above 80 points.

The performance score of conjugated material flutes is higher, and conjugated material flutes usually have richer and warmer timbres, which can better express the delicate emotions of music.

Flute made of ceramic and metal materials may have a relatively sharp or hard tone quality due to the characteristics of the material. This difference may affect the performer’s ability to express music and reduce training effectiveness.

Flutes made of different materials have differences in acoustic characteristics. Conjugated materials can produce better sound effects and resonance, which helps to improve the richness of timbre and the stability of intonation. In contrast, ceramic and metal flutes may lack this resonance effect, which affects the performance of the training effect.

### 4.5 User experience

**FIGURE 6:**
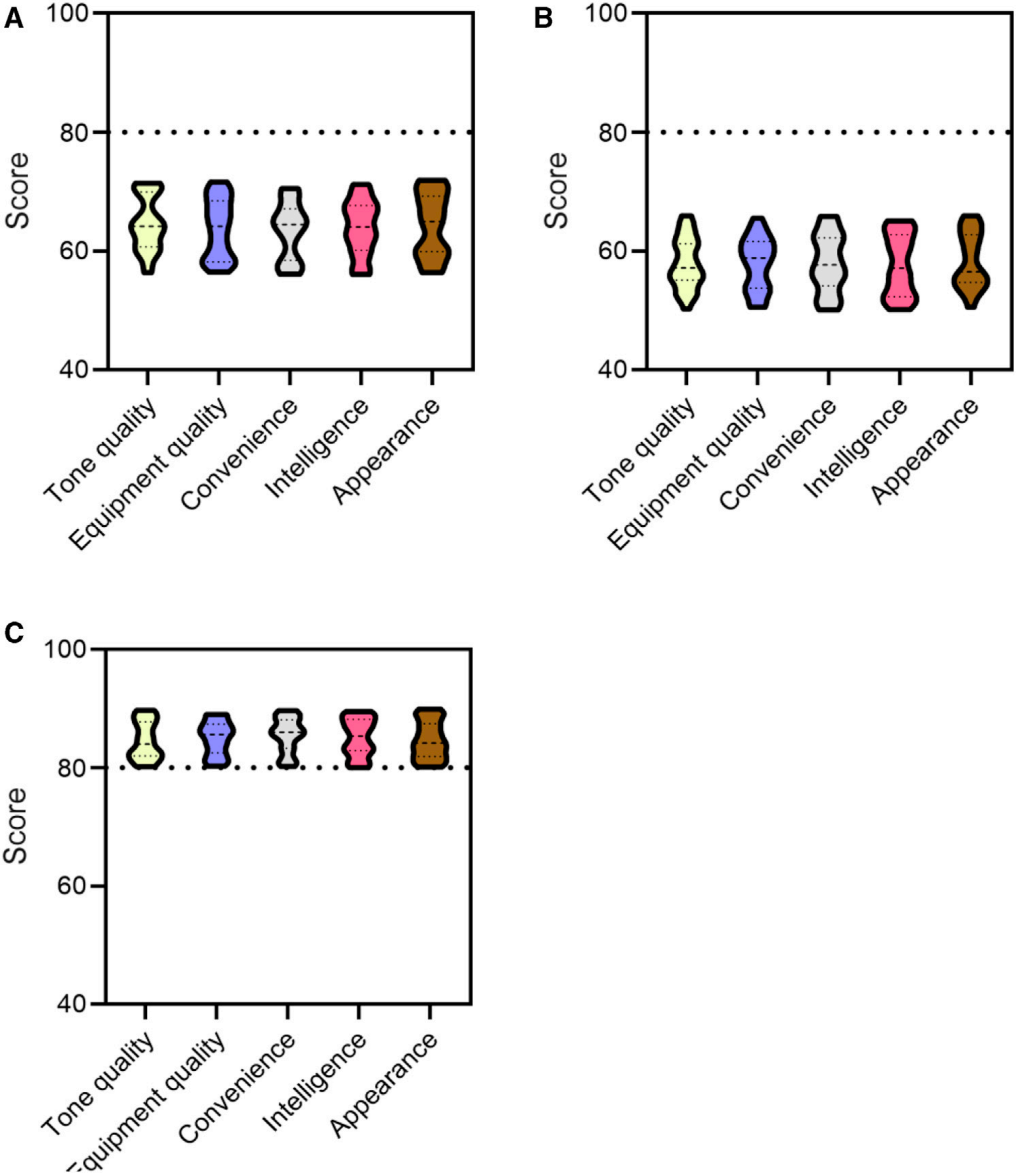
User experience rating of flutes made of different materials. **(A)** Experience rating of ceramic flute. **(B)** Experience rating of metal flute. **(C)** Experience rating of conjugated material flutes.

Through feedback from 30 performers, the user experience of the equipment was evaluated, with indicators including tone quality, equipment quality, convenience, intelligence, and appearance. The user experience ratings of different materials of flutes are shown in Figure 6 (in Figure 6, the horizontal axis represents tone quality, equipment quality, convenience, intelligence, and appearance, while the vertical axis represents ratings).

As shown in Figure 6: Figure 6A showed the experience rating of 30 performers on ceramic flutes, with ratings for tone quality, equipment quality, convenience, intelligence, and appearance all below 80 points; Figure 6B showed the experience ratings of 30 performers on metal flutes, with ratings for tone quality, equipment quality, convenience, intelligence, and appearance all below 80 points; Figure 6C showed the experience rating of 30 performers on conjugated material flutes, with an overall rating of over 80 for tone quality, equipment quality, convenience, intelligence, and appearance.

The overall score of conjugated material flutes is higher than that of ceramic and metal flutes, and the flexibility of conjugated materials allows performers to create rich musical effects.

The tone quality of flutes made of ceramic and metal materials are different from those made of conjugated materials. Conjugated materials generally have good acoustic properties, can emit rich and soft sounds, and exhibit high tone quality. In contrast, flutes made of ceramic and metal have a somewhat monotonous and sharp tone, which cannot be performed at a high level like conjugated material flutes.

## 5 Discussion

Figure 4 shows that the playing error rate of the conjugated material flute is lower. Because ceramics and metals may have issues such as energy attenuation, signal distortion, or resonance during audio transmission, resulting in an increase in misjudgment rates. Conjugated materials have good vibration reduction performance and can effectively suppress disordered vibration and external interference. In this way, the flute made of conjugated materials can accurately capture and identify the music being played by the performer, reducing the rate of misjudgment. Conjugated materials have strong noise resistance and can effectively reduce the interference of external noise. This can distinguish the sound played by the performer from the surrounding noise, thereby reducing the rate of misjudgment. The rapid response of conjugated materials can better capture and identify the music that the performer wants to play. In contrast, ceramics and metals may experience significant delays during the response process, which can affect the accuracy and misjudgment rate of detection results.

Table 3 shows that the average time delay of conjugated material flutes is lower because conjugated materials can better transmit and amplify acoustic energy, thereby improving sensitivity. The sound conductivity of different materials also has a certain impact on the sensitivity of the flute. The fibrous structure of conjugated materials enables them to propagate better in the air, thereby obtaining higher quality sound sources. However, ceramics and metals can cause energy loss or attenuation, which affects the sensitivity of sensors.

Due to the relatively high hardness of ceramics and metals, they are subjected to high stress and energy consumption. When the performer plays into this hole, a portion of it is consumed without being converted into sound, resulting in a decrease in the vibration effect and sensitivity of the material. Flutes made of conjugated materials are generally hollow, and with thin-walled tubes, they can better respond to the player’s subtle movements. In contrast, ceramic and metal flutes are made of sturdy materials, with thicker pipe walls and heavier structures. This structural difference has an impact on vibration and sensitivity.

Figure 5 shows that the performance of the conjugated material flute is better. Due to its structural and material characteristics, conjugated material flutes typically provide better feedback and sensitivity, helping performers’ better grasp elements such as pitch, timbre, and volume. In contrast, ceramic and metal flutes may not be sensitive enough to accurately perceive and adjust subtle playing techniques and performance.

## 6 Conclusion

Due to the unique intelligent sensing effect of conjugated materials, they have been widely used in many fields. In the production of musical instruments, the use of conjugated materials can provide them with flexible sensing functions, improve their tone quality, and provide new possibilities for the production of musical instruments. Therefore, this new type of conjugated material has good application prospects in the production of musical instruments. By utilizing the intelligent sensing function of conjugated materials, a flute practice equipment with intelligent characteristics can be produced, which can greatly improve the practice effect and efficiency of students. In the future, with the development of technology, intelligent sensing equipment would play a more important role in music education, allowing more students to feel the joy and satisfaction of learning musical instruments.

## Data Availability

The original contributions presented in the study are included in the article/Supplementary Material, further inquiries can be directed to the corresponding author.
